# Hormonal therapies and venous thrombosis: Estrogen matters!

**DOI:** 10.1016/j.rpth.2022.100021

**Published:** 2023-02-15

**Authors:** Laure Morimont, Mitchell D. Creinin, Ulysse Gaspard, Jean-Michel Foidart, Jonathan Douxfils

**Affiliations:** 1 QUALIblood s.a., Namur, Belgium; 2 Faculty of Medicine, Department of Pharmacy, Namur Research Institute for Life Sciences, Namur Thrombosis and Hemostasis Center, Clinical Pharmacology Research Group, University of Namur, Namur, Belgium; 3 Department of Obstetrics and Gynecology, University of California, Davis, Sacramento, California, USA; 4 Department of Obstetrics and Gynecology, University of Liège, Liège, Belgium; 5 Estetra srl, Liège, Belgium; 6 University of Liège, Liège, Belgium

To the Editor,

We read the article by LaVasseur et al. on hormonal therapies and venous thromboembolism (VTE) and are concerned about the lack of discrimination between different marketed estrogens and a lack of understanding of progestins.

First, the authors used “progesterone” rather than “progestogen,” which is not the correct nomenclature according to the terms recommended by the North American Menopause Society [1]. Progesterone is a natural progestogen, whereas progestins are synthetic progestogens. Misclassification of hormones causes misunderstanding of their effects because important differences exist between progestins based on numerous cellular, biological, and clinical effects [2].

Second, classification of combined oral contraceptives (COCs) based on generations is inappropriate, especially while discussing the risk of VTE. The total estrogenicity of the preparation should be the concern of physicians rather than the “generation” of the associated progestin [2]. Although some ethinylestradiol (EE)-containing pills are classified as combinations with the highest risk of VTE, estradiol (E2)-containing pills showed a pooled crude hazard ratio of 0.73 (95% CI, 0.41-1.29) versus that of EE/levonorgestrel (LNG), and considering the age-related increased risk of VTE, these results are considerable because women using E2-based COCs were 2 to 6 years older than those in the group using EE/LNG [3].

Third, the authors misclassified the recently approved estetrol (E4)/drospirenone (DRSP) combination as a preparation with a risk of VTE similar to that of EE/DRSP. E4, a natural estrogen, has a pharmacologic profile distinct from that of EE and E2, as summarized by Gerard et al. [4]. Data obtained during the development of 15-mg E4/3-mg DRSP demonstrated minimal impact on coagulation parameters compared to 20-μg EE /3-mg DRSP and 30-μg EE/150-μg LNG (Figure B). The incidence of VTE with E4/DRSP in the phase 3 study was 3.7 of 10,000 women-year, with only 1 case of VTE among 3417 participants (United States/Canada and European/Russian studies). Although these studies were not designed to investigate the risk of VTE and not powered for that particular outcome, the fact that only 1 case of VTE occurred in 3,417 women is reassuring and suggests a lower risk of VTE than with EE-containing products. In comparison, the number of VTE cases in other contraceptive United States trials was 3 for 10-μg EE/1-mg norethindrone acetate (N = 1,683), 4 for a vaginal ring delivering 13-μg EE and 150-μg segesterone acetate per day (N = 1,188), and 4 for a patch with dosing equivalent to a 30-μg EE /120-μg LNG COC (N = 2,031) [4].

**Figure:**
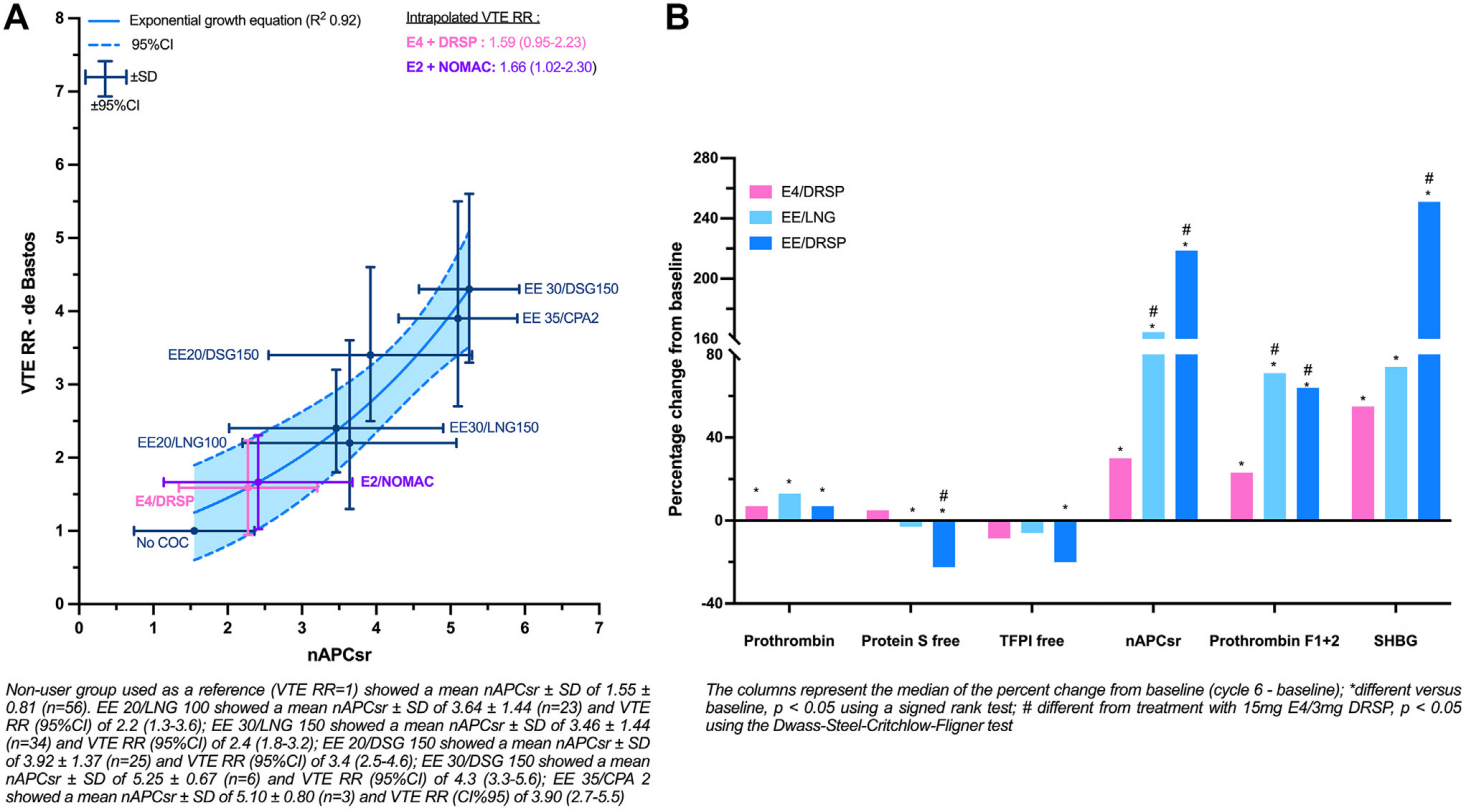
(A) Association between normalized activated protein C sensitivity ratio data and relative risk of venous thromboembolism in women using combined oral contraceptives and (B) comparison of the impact of E4/DRSP versus that of EE/LNG and EE/DRSP on key coagulation markers. COC, combined oral contraceptive; CPA, cyproterone acetate; DRSP, drospirenone; DSG, desogestrel; E2, estradiol; E4, estetrol; EE, ethinylestradiol; LNG, levonorgestrel; nAPCsr, normalized activated protein C sensitivity ratio; NOMAC, nomegestrol acetate; RR, relative risk; SHBG, sex hormone-binding globulin; TFPI, tissue factor pathway inhibitor; VTE, venous thromboembolism.

These clinical data are also supported by an *in silico* model investigating the association between the normalized activated protein C sensitivity ratio (nAPCsr) and the relative risk (RR) of VTE in COC users (Figure A). In this model, independent nAPCsr data were obtained in 237 women taking different COCs and grouped according to the COC. The RR of VTE with a particular COC versus that of VTE in nonusers was extracted from the Cochrane meta-analysis of de Bastos et al. [5] when available (dark blue points in Figure A). Based on these correlation points, we were able to generate a model reflecting the association between mean nAPCsr obtained in a population treated with a particular COC and the RR of VTE of that COC. Then, by interpolating the nAPCsr data obtained with 15-mg E4/3-mg DRSP, we were able to estimate that the RR of VTE versus that of VTE in nonusers was 1.59 (95% CI, 0.95-2.23). Indirect comparison with 20-μg EE/100-μg LNG provided an RR of 0.72 (95% CI, 0.43-1.01). External validation of the model has been performed since the estimated RR of VTE with 1.5-mg E2/2.5-mg nomegestrol acetate was estimated as being 1.66 (95% CI, 1.02-2.30) versus that of nonusers, an estimation that is consistent with the adjusted RR of VTE of the prospective surveillance study of 1.5-mg E2/2.5-mg nomegestrol acetate (RR, 1.63; 95% CI, 0.63-4.19).

To conclude, although this review contains some valuable insight, it is not completely in line with the contemporary understanding of hormones, especially because they relate to the risk of VTE.

## References

[1] Stanczyk F.Z., Hapgood J.P., Winer S., Mishell D.R. (2013). Progestogens used in postmenopausal hormone therapy: differences in their pharmacological properties, intracellular actions, and clinical effects. Endocr Rev.

[2] Creinin M.D., Jensen J.T. (2020). Oral contraceptive generations—time to stop using a marketing myth to define nomenclature. Contraception.

[3] Grandi G., Facchinetti F., Bitzer J. (2022). Confirmation of the safety of combined oral contraceptives containing oestradiol on the risk of venous thromboembolism. Eur J Contracept Reprod Health Care.

[4] Gerard C., Arnal J.F., Jost M., Douxfils J., Lenfant F., Fontaine C. (2022). Profile of estetrol, a promising native estrogen for oral contraception and the relief of climacteric symptoms of menopause. Expert Rev Clin Pharmacol.

[5] de Bastos M., Stegeman B.H., Rosendaal F.R., Vlieg A.V., Helmerhorst F.M., Stijnen T. (2014). Combined oral contraceptives: venous thrombosis. Cochrane Database Syst Rev.

